# Biting the hand that feeds: Metabolic determinants of cell fate during infection

**DOI:** 10.3389/fimmu.2022.923024

**Published:** 2022-10-13

**Authors:** Isabella Fraschilla, Charles L. Evavold

**Affiliations:** ^1^ Program in Immunology, Harvard Medical School, Boston, MA, United States; ^2^ Ragon Institute of MGH, MIT and Harvard, Cambridge, MA, United States

**Keywords:** pathogenesis, cell-intrinsic immunity, inflammasome, inflammation, guard theory, metabolism, host-pathogen arms race, cell fate decisions

## Abstract

Metabolic shifts can occur in cells of the innate immune system in response to microbial infection. Whether these metabolic shifts benefit host defense and propagation of an immune response appears to be context dependent. In an arms race, host-adapted microbes and mammalian cells vie for control of biosynthetic machinery, organelles, and metabolites. Herein, we discuss the intersection of host metabolism and cell-intrinsic immunity with implications for cell fate during infection. Sensation of microbial ligands in isolation results in host metabolic shifts that imbues normal innate immune function, such as cytokine secretion. However, living microbes have an arsenal of effectors and strategies to subvert cell-intrinsic immune responses by manipulating host metabolism. Consequently, host metabolism is monitored as an indicator of invasion or manipulation by a pathogen, primarily through the actions of guard proteins and inflammasome pathways. In this review, we frame initiation of cell-intrinsic immunity in the context of host metabolism to include a physiologic “Goldilocks zone” of allowable shifts with guard circuits monitoring wide perturbations away from this zone for the initiation of innate immune responses. Through comparison of studies with purified microbial ligands, dead microbes, and live pathogens we may begin to understand how shifts in metabolism determine the outcome of host-pathogen interactions.

## Introduction

Survival of microbes and host organisms during infection depends on the acquisition of limited basic nutrients. Through catabolic and anabolic cellular chemistries that are broadly termed metabolism, host cells and microbes can generate energy equivalents, biosynthetic intermediates, macromolecules, and signaling moieties. From an evolutionary standpoint, plasticity in metabolic inputs and outputs can be viewed as beneficial to host and microbial fitness. For example, certain pathogenic strains of bacteria have evolved unique metabolic enzymes to outcompete their commensal counterparts for nutrients and colonize the intestinal lumen (1). While host and pathogens are under evolutionary pressure to develop immune strategies and virulence effectors to counter each other (2), metabolic pathways are also likely under selective pressure in this arms race. For microbes, metabolic manipulation is required for replication, dissemination, and subversion of the immune response (3). On the host side, metabolic shifts can be induced to modulate cell-intrinsic and tissue-level host defense (4, 5). Beyond direct detection of microbial ligands, host metabolic pathways are also monitored as a central node in pathogen detection, as extreme metabolic perturbations are a sign of infection and danger to the host (6–8). Despite the essentiality of bacterial metabolism for competitive niche occupancy (1), metabolite-responsive gene programs, particularly those that control virulence factors, remain unexplored in many pathogens. Bioinformatics-based mapping of enzymes encoded by bacterial genomes and consideration of the metabolic signals present at sites of infection can inform immunologists on previously underappreciated aspects of pathogenesis and the infective life cycle of host-adapted microorganisms.

**Figure 1:**
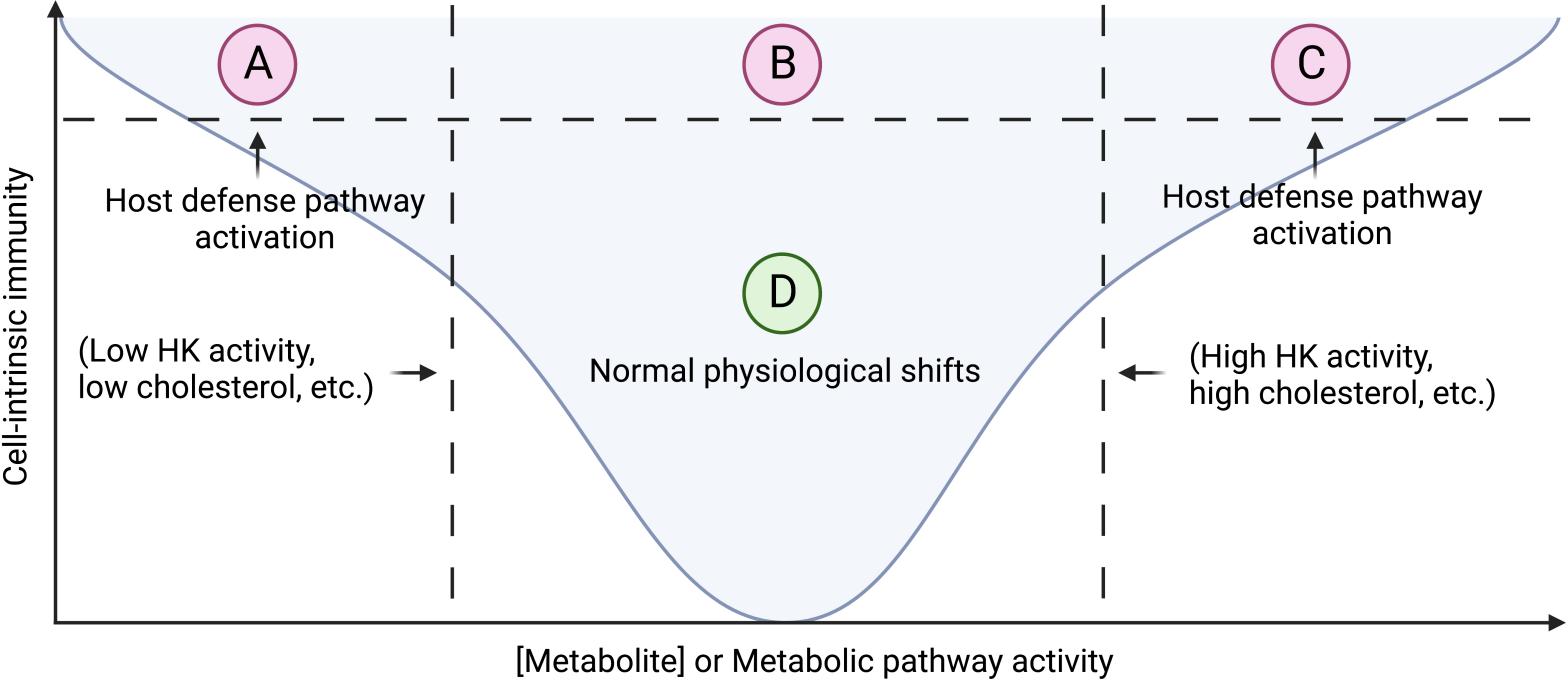
A Goldilocks zone of physiologic shifts in metabolic activity with guard circuits surveying wide perturbations. **(A)** Low concentration of a monitored metabolite or metabolic activity can lead to increased cell-intrinsic immunity through inflammasome activation in the case of low hexokinase (HK) activity or IFN production and ISG expression in low cholesterol biosynthesis. **(B)** Physiological range of host-adaptive changes in metabolite concentration and metabolic activity does not preclude cell-intrinsic immunity through orthogonal inflammasome activation such as monitoring for direct microbial ligands. **(C)** High concentration of a monitored metabolite or metabolic activity can lead to increased cell-intrinsic immunity through inflammasome activation in the case of high hexokinase (HK) activity or cholesterol accumulation. **(D)** A Goldilocks zone of allowed host-adaptive changes in metabolite concentration and metabolic activity without incurring cell-intrinsic immune responses such as inflammasome activation.

First formulated to describe plant innate immune responses, the guard protein model of cell-intrinsic immunity posits that pathogens can be detected through indirectly monitoring for host manipulation by microbes (7, 9). This defense strategy can be accomplished by monitoring key cellular pathways to check the activity of a component of a given pathway. Examples of processes that are monitored by innate immune pathways for indication of pathogen invasion include organelle and cytoskeleton homeostasis, canonical signal transduction pathways, and metabolic pathways (7). One facet of innate immune cell fate involves epigenetic states recently termed “trained immunity” that encapsulates the intersection between pattern recognition receptor signaling, metabolic rewiring, and epigenetic adaptations to stimuli that we consider beyond the scope of this review and have been expertly discussed elsewhere (10–13). In this review, we will focus on innate immune monitoring of host metabolic pathways. While pattern recognition receptors (PRRs) can directly sense microbial products to mediate transcriptional and post-translational induction of cell-intrinsic immunity (6), we will consider how direct detection of pathogen associated molecular patterns (PAMPs) or damage associated molecular patterns (DAMPs) may influence host metabolism (independent of transcriptional and translation activities within the cell). Alternatively, guard circuits monitor for patterns of pathogenesis as might be ascertained through cellular readouts of metabolic pathway activity, such as accumulation or depletion of metabolites. While our current understanding of metabolic monitoring derives from experiments using select pathogens, we hypothesize that this feature of innate immunity is generalizable to other microbial infections that subvert host metabolism during pathogenesis. We take a particular interest in metabolic shifts associated with classically and alternatively activated macrophage cell fates in dictating pathogen replication and host defense. We also consider metabolic monitoring from the perspective of inflammasome signaling as a prototypical cell-intrinsic defense program. As primarily cytosolic signaling complexes, inflammasomes mediate cell-intrinsic immunity through various means with hallmarks including inflammatory caspase activation, cleavage and release of IL-1β, and membrane pore formation and cellular lysis mediated by gasdermin D (GSDMD) pores (13–15). As inflammasome activation can have drastic consequences for cellular viability and is highly inflammatory locally and systemically, we suggest that a Goldilocks zone permits physiologic shifts in metabolic pathways and metabolites that accompany adaptation to environmental nutrients and stressors. When pathogens manipulate metabolic outputs for their own purposes, these deviations from the Goldilocks zone may be sensed by guard pathways that initiate host defense strategies, including but not limited to cell-intrinsic immune responses *via* inflammasomes (**Figure 1**).

## Monitoring host metabolism as an innate immune strategy

**Figure 2:**
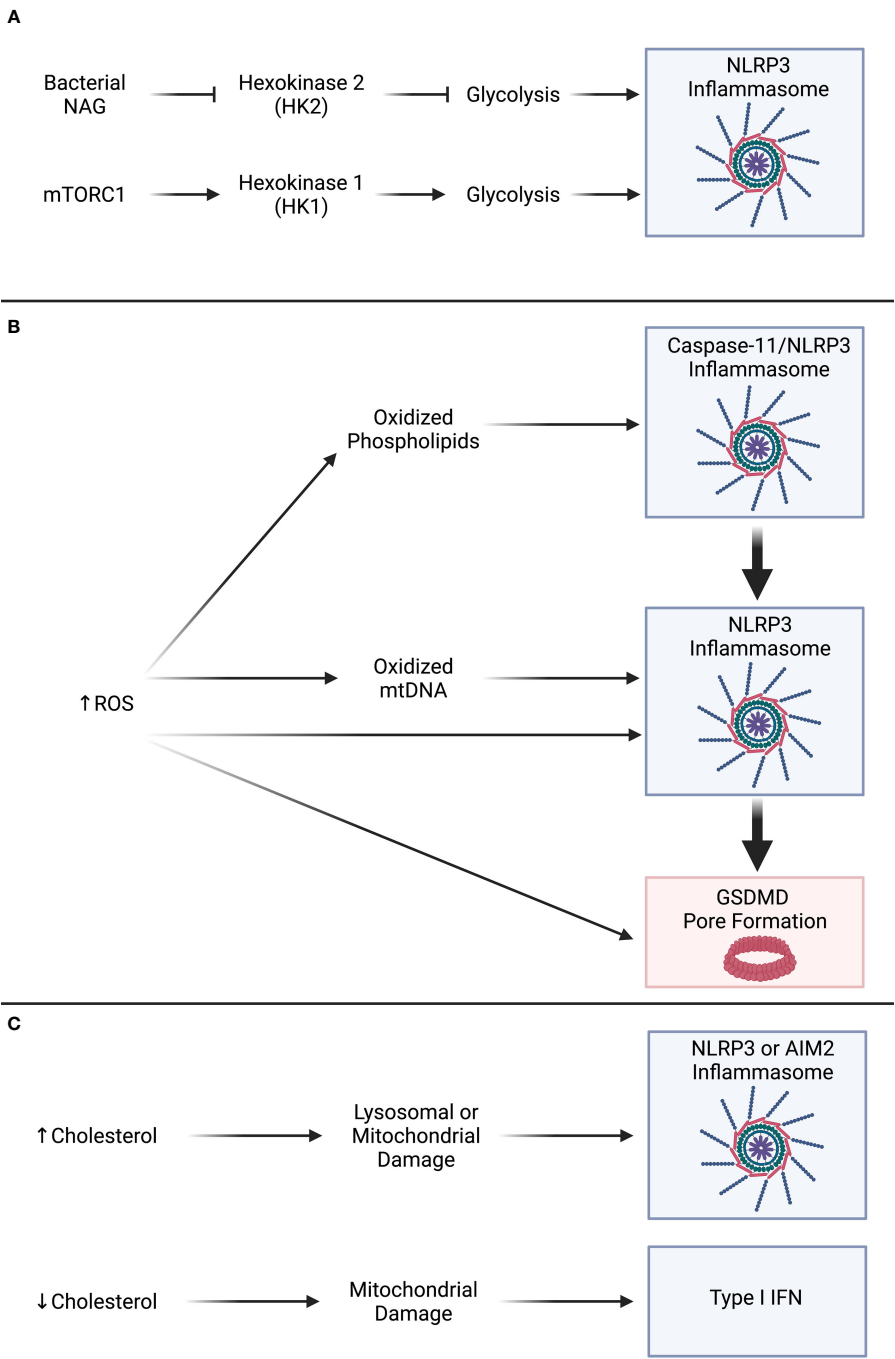
Guard circuits sense metabolic perturbations and activate inflammasome pathways. **(A)** Inhibition of glycolysis *via* hexokinase 2 (HK2) recognition of bacterial N-acetyl glucosamine (NAG) or activation of glycolysis *via* increased hexokinase 1 (HK1) activity result in NLRP3 inflammasome activation. **(B)** Increased production of reactive oxygen species (ROS) metabolites activates the NLRP3 inflammasome, such as through the generation of oxidized lipids, and directly promotes gasdermin D (GSDMD) pore formation. **(C)** Alterations to homeostatic cholesterol levels can result in lysosomal or mitochondrial dysfunction that results in inflammasome activation or type I interferon (IFN) production.

Here, our first example of a host guarding circuit involves the metabolic enzyme hexokinase, which normally acts as commitment step for host glucose metabolism by phosphorylating cytosolic pools of glucose to glucose-6-phosphate (16). Seminal work demonstrated that hexokinase 2 (HK2) also recognizes N-acetyl glucosamine (NAG) sugars derived from the degradation of Gram-positive bacterial peptidoglycan (PGN) (17). However, this microbial recognition inactivates HK2, ultimately resulting in the activation of the protein NLRP3, which then seeds the assembly of the NLRP3 inflammasome (**Figure 2**). Treatment of macrophages with chemical inhibitors of HK2, such as 2-DG, were able to recapitulate NLRP3 inflammasome activation (17). Inflammasome activation in murine bone marrow derived macrophages (BMDMs) infected with mutant strains of *Staphylococcus aureus* with highly labile peptidoglycan (PGN) also stimulates inflammasome activity as seen by the induction of GSDMD pore formation and IL-1β secretion that notably does not proceed to pyroptotic lysis in a process termed hyperactivation (17–19). Moreover, stimulation of BMDMs with particulate PGN from various gram-positive bacteria and direct transfection of purified NAG sugars results in NLRP3 inflammasome activation and phagocyte hyperactivation. Therefore, we posit that HK acts as a guardee within a metabolic guard pathway where an undiscovered intermediate or NLRP3 itself is the guard that activates a cell-intrinsic immune response. As glycolysis and productive HK activity have also been demonstrated to be necessary for NLRP3 inflammasome activity in other contexts (20), more work is needed to uncover if inflammasomes or other innate immune pathways survey perturbations from homeostasis whereby guard proteins are also relatively more active. Analogous paradoxical control of inflammasome signaling has recently been noted regarding the role of electron transport chain (ETC) inhibition in prevention of NLRP3 inflammasome activation that could be rescued by maintenance of ATP levels through orthogonal means (21), whereas seminal work suggests that inhibition of the ETC at complex I and III promote ROS production and NLRP3 activation (22). ROS may also act at the stage of transcriptional priming of inflammasome components including the receptor NLRP3 (23). The observation that inhibition or activation of the same metabolic pathway can activate inflammasomes suggests that hexokinase-mediated metabolism is subject to innate immune surveillance for deviation from a Goldilocks zone.

Other microbial ligands, such as bacterial cell wall lipopolysaccharides (LPS) from Gram-negative bacteria, can stimulate inflammasome activation and cellular hyperactivation in human monocytes (24). Whether monocyte hyperactivation *via* LPS-treatment depends on deviation from a metabolic Goldilocks zone is currently unknown, but recent work has determined that treatment of monocytes with several concurrent PAMPs, including LPS, can shift normally hyperactive monocytes towards a pyroptotic phenotype likely through increased activity of the pore forming protein GSDMD and increased generation of reactive oxygen species (ROS) metabolites (25). Recent work determined that the Ragulator-Rag-mTORC1 metabolic axis can promote pyroptosis specifically at the stage of GSDMD oligomerization (26). Ragulator-Rag appears to control a portion of cellular ROS production in macrophages that may be indicative of a necessary byproduct of host metabolic activities, such as respiration (26–29). Bolstering a causal relationship between GSDMD oligomerization and ROS metabolites, further investigation demonstrated that bacterial LPS or fungal β-glucans can induce ROS in parallel to the Ragulator-Rag pathway to promote GSDMD pore formation and pyroptosis (30). These recent data and former studies suggest that diverse sources of ROS metabolites can feed into the inflammasome pathway at various stages to promote or inhibit inflammasome activities (22, 23, 26, 30–32) (**Figure 2**). As ROS production often coincides with mitochondrial dysfunction or microbial infection, monitoring of cellular redox state and sensation of ROS metabolites by innate immune guard proteins may contextualize danger to the host. Moreover, host-derived DAMPs that may indicate immune- or pathogen-induced tissue damage can induce cellular hyperactivation in human and mouse dendritic cells and mouse macrophages (19, 33, 34).

ROS metabolites can react with other molecules, such as lipids within cellular and microbial membranes, in addition to its signaling or damaging roles towards biological macromolecules, such as proteins and nucleic acid polymers (35–38). Indeed, oxidation of host lipids that contain double bonds in their acyl chains results in generation of bioactive metabolites that are sensed by the innate immune system and can also serve as alternative metabolic inputs for fueling cellular metabolism (39–42). One such example is oxidation of lipids in low density lipoprotein (LDL) particles in atherosclerosis or oxidation of phospholipids found in host cell membranes during neutrophil responses to damage or pathogen infection in the lung (39, 40, 43–45). These lipids, when exposed to ROS, can generate an amalgam of bioactive oxidized lipids termed oxidized PAPC (oxPAPC) (33, 39, 40). Thus, oxidized lipids are indicative of host metabolic dysfunction and damage to host cells as occurs in organismal metabolic disorders or during an immune response against invading pathogens (43–45) (**Figure 2**). Some studies have determined that oxPAPC can serve as a competitive inhibitor for innate immune signaling pathways as they are structurally similar to bacterial LPS. In certain contexts, oxPAPC can buffer the activities of bacterial LPS during activation of pro-inflammatory transcription downstream of TLR4 signaling or pyroptosis downstream of caspase-11 inflammasome activation (46–49). Recent work has also determined that oxPAPC can lead to dendritic cell hyperactivation through sub-lytic GSDMD pore formation and secretion of bioactive IL-1β without pyroptotic lysis (19, 33, 34, 50). Moreover, macrophages can also become hyperactivated by treatment with a microbial PAMP followed by subsequent treatment with the bioactive constituents of oxPAPC, namely PGPC and POVPC (19, 51). Cells that have been hyperactivated in response to oxidized lipids also display unique metabolic features including maintenance of mitochondrial activities and differential energy usage through glutaminolysis (41). Furthermore, ROS may intersect with the NLRP3 inflammasome activation through the oxidation of newly generated mitochondrial DNA in a process that may suggest oxidized DNA can serve as a ligand for NLRP3 (52–54). Calcium (Ca) flux is a known determinant of mitochondrial ROS production (55). Recent work also suggests that Ca mediates the release of oxidized mitochondrial DNA through mitochondrial permeability transition pores and the oligomerization of VDAC channels to control NLRP3 activation (56). These examples demonstrate host inflammasomes survey for signs of metabolic dysfunction and pathogen invasion by monitoring metabolic pathways, organelles, or damage-associated metabolites and secondary messengers.

## Amino acid availability and protein synthesis

Host cells monitor amino acid availability through two major metabolic arms, the Ragulator-Rag-mTORC1 axis and eIF2α- and GCN2-mediated monitoring of translation and tRNA charged status (57, 58). Amino acid depletion may thus serve as a conserved contextual signal of pathogenic invasion as microbes, such as bacteria, may utilize amino acids as energy sources and building blocks for their own proteins and cell wall macromolecules (3). Moreover, viruses must take over host translation machinery for generation of viral polypeptides and new viral particles during replication (59). As translational output is related to amino acid availability, we also consider translation and protein synthesis as a potential indicator of pathogen manipulation that can intersect with cell-intrinsic immunity (58, 60).

mTORC1 is a major complex involved in broad metabolic regulation of the host cell as a switch whose kinase activity determines anabolic and catabolic processes through specific substrate recruitment (61). mTORC1 phosphorylates the anabolic targets 4E-BP1 and S6 kinase 1 to promote host translation (61). ULK1 and TFEB are catabolic regulators whose functions are repressed by mTORC1 (57). mTORC1 phosphorylation of ULK1 can directly repress autophagic activity (57, 61). Moreover, TFEB is the master transcriptional regulator of genes involved in lysosome biogenesis and autophagy (62, 63). Phosphorylation of TFEB by mTORC1 retains TFEB in the cytosol through interaction with 14-3-3 proteins (64, 65). When mTORC1 activity is diminished, such as during some pathogen infections or other nutrient depleted settings, TFEB is unleashed to translocate to the nucleus to transcriptionally activate autophagy and lysosome-dependent degradative processes to recycle host macromolecules and nutrients (63, 66). Amino acid availability is monitored by regulators of mTORC1, such as the lysosomal transporter SLC38A9, the vacuolar ATPase (v-ATPase that tethers Ragulator-Rag to the lysosome), and Ragulator-Rag (57, 61). Moreover, Ragulator-Rag is a specific regulator of TFEB as itdirectly recruits TFEB to mTORC1 for repressive phosphorylation under amino acid replete contexts (67, 68). Beyond monitoring of cytosolic and lysosomal stores of free amino acids by Sestrins, CASTOR proteins, and Ragulator-Rag to control mTORC1 activity, amino acid availability can also be monitored indirectly by the kinase GCN2 (57, 58, 61). When uncharged tRNAs accumulate, GCN2 binds to uncharged tRNAs to phosphorylate the translation regulator eIF2α to shut down translation (58).

Pathogen infection can be sensed through perturbations of amino acid availability (69). In the context of bacterial infection, amino acids can be consumed or mislocalized within the host through the action of microbial effectors and metabolism (7, 69). Intracellular bacteria may deplete or mislocalize amino acid pools *via* perforation of endomembranes, such as the lysosome (3, 69). Amino acid depletion can inactivate the Ragulator-Rag-mTORC1 axis and thus promote autophagy directly through ULK1 and the transcriptional activity of TFEB (57, 61). Autophagy of bacteria, also termed xenophagy, is a host defense strategy to capture cytosol invading bacteria and target them for ultimate degradation in lysosomes (69). Consistent with a pathway of membrane perforation, amino acid depletion (or potentially mislocalization) and activation of host protective xenophagy, membrane damaging effectors like the *Listeria monocytogenes* pore forming toxin LLO can activate xenophagy (70). Moreover, membrane perforation and delivery of bacterial effectors through syringe-like secretion systems, such as the Type III secretion systems of *Shigella flexneri* and *Salmonella enterica* serovar Typhimurium, also trigger xenophagy (71). Bacteria-induced endomembrane damage and delivery of effectors can also activate the GCN2 arm of amino acid monitoring (71, 72). Viral infection can also be detected through modulation of amino acid availability, as the eIF2alpha kinases GCN2 and protein kinase R (PKR) are required for the induction of autophagy in response to infection with herpes simplex virus 1 (HSV-1) (73). Highlighting an evolutionary host defense pressure, HSV-1 encodes a virulence product that antagonizes autophagy induction (73). Human immunodeficiency virus (HIV) can also manipulate the GCN2 pathway to suppress host translation in favor of viral replication early in infection (74). This may suggest that GCN2 and PKR control of translation and autophagy may antagonize viral infections more generally.

A careful mechanistic examination into how membrane permeability might lead to depletion of amino acids is needed. Experiments utilizing lysomotrophic damaging peptides such as L-leucyl-L-leucine methyl ester (LLOMe) have also demonstrated that an organelle homeostasis and endomembrane monitoring system exists (75, 76). This system employs ESCRT-III machinery presumably for membrane repair or degradation of damaged membrane sections through multivesicular body production analogous to membrane repair activities that occur at the plasma membrane (76, 77). For a larger magnitude of membrane or organelle damage, a galectin-Ragulator-Rag dependent mechanism is activated (75, 76). Endomembrane damage may lead to long-term depletion of amino acids through inhibited catabolism. We speculate that a burst of amino acids may also have evolved a sensor as an indication of danger or damage to the host. Whether amino acid monitoring pathways such as Ragulator-Rag can respond to increases in amino acid concentration beyond physiological levels is currently unknown. The Ragulator-Rag pathway can also respond to mitochondrial dysfunction presumably to activate mitophagy of damaged mitochondria (78). Therefore, Ragulator-Rag may serve as a master regulator of endomembranes and organelle homeostasis through integration of host metabolic state and amino acid pools.

As suggested by study of pathogen activation or evolved subversion of GCN2 and Ragulator-Rag-mTORC1, protein translation and dysfunction may serve as a contextual cue of pathogen invasion or manipulation of the host (60, 69). We will consider the monitoring of the unfolded protein response (UPR) and endoplasmic reticulum (ER) stress as a host defense strategy (7, 60). Several intracellular bacteria utilize ER-derived compartments for replication such as *Legionella pneumophila* (3, 79, 80). Moreover, viruses often remodel ER and mitochondrial membrane sites as replication sites for production of new viral particles (3, 59). *Legionella* encodes a type IV secretion system to remodel the ER compartment for its replicative needs (81, 82). Consequently, UPR can become activated after bacterial and viral infection (83–85). *Legionella* also encodes type IV secretion system delivered effectors that appear to limit host translation that may suggest subversion of ER stress (81, 82, 86). Recent work provides direct evidence that inhibition of host translation using vesicular stomatitis virus infection and chemical inhibitors as models can activate cell-intrinsic immunity through caspase-3 and GSDME dependent pyroptosis. In this pathway, labile members of the BCL2 family with high constitutive turnover act as a guard circuit to monitor changes in translation output and initiate cell death (87). HSV-1 has evolved mechanisms to subvert the activation of caspase-3 and subsequent GSDME-mediated pyroptosis through the action of the protein ICP27 (87). Prior studies also suggest that ER stress can induce secretion of cleaved IL-1β and pyroptosis through diverse mechanisms (88, 89). Downregulation of host translation may further subvert immune responses at large that require *de novo* translation of pro-inflammatory cytokines or cell-intrinsic defense proteins, but this translation shutdown may come with the cost of induction of cell-intrinsic immune programs such as pyroptosis in certain contexts.

## Cholesterol and innate immunity

Monitoring of central host metabolites or biosynthetic pathways could serve as a strategy to detect invasion or manipulation of the host by pathogenic microbes. A prime example of internal monitoring of host metabolite deviation for activation of innate immune responses is the monitoring of cholesterol content and biosynthesis (90). Most microbes are not thought to generate their own cholesterol though some can incorporate cholesterol into viral envelopes or bacterial cellular membranes (3, 59, 91). Moreover, cholesterol is thought to be consumed by some species of bacteria, such as *Mycobacterium tuberculosis* (92–94). Cholesterol is a major component of host cell membranes and as such microbes have evolved ways to utilize the presence of cholesterol for targeting of toxins, microbial entry into the cell, and as fuel sources (90, 91). Necessarily, the host has also evolved mechanisms to modulate the abundance and location of cholesterol stores to decrease susceptibility to intoxication and infection (90). As with other examples provided above, monitoring of cellular cholesterol may represent an example of a Goldilocks guarding circuit as distinct cell-intrinsic immune responses are triggered by diminished cholesterol biosynthesis as well as cholesterol overload (**Figure 2**).

Host cholesterol is derived from exogenous sources through extracellular uptake or *de novo* biosynthesis (95). Cholesterol found within extracellular low-density lipoprotein (LDL) can be internalized by host cells through the action of endocytosis downstream of the LDL receptor (LDLR) (95). Once cholesterol is in the lysosome, it can be imported into cellular membrane pools *via* NPC1 and NPC2 (96). Alternatively, cholesterol can be synthesized within host cells through the action of genes under the transcriptional control of the master regulator SREBP2 (97). Notably, LDLR is also under transcriptional control of SREBP2 (95). In addition to being incorporated into host membranes, cholesterol can be stored within lipid droplets within cells or secreted from cells through efflux pathways whereby the intracellular concentration of cholesterol is adaptively maintained in a Goldilocks zone (95).

During organismal metabolic dysfunction, cholesterol can accumulate to high concentrations and form cholesterol crystals that damage lysosomes (98). Lysosomal damage in response to cholesterol accumulation results in activation of the NLRP3 inflammasome, secretion of bioactive IL-1β, and pyroptotic cell death (98). An inflammasome seeded by the protein AIM2, which binds directly to cytosolic DNA, can be activated in response to mitochondrial DNA (mtDNA) (99). Type I interferons (IFNs) and production of the metabolite 25-hydroxycholesterol (25-HC) can inhibit cholesterol synthesis and limit AIM2 inflammasome activation likely through restraining cholesterol driven mitochondrial dysfunction (99, 100). While these studies demonstrate that type I IFNs can restrain AIM2 activation likely at the level of endogenous host-derived ligands such as mitochondrial DNA, AIM2 is an IFN stimulated gene (ISG) and thus transcriptionally induced by type I IFN signaling that can promote sensation of pathogenic DNA from bacteria and viruses in the host cytosol (101–103). Further work suggests that diminished cholesterol biosynthesis can also result in a cell-intrinsic immune response through the production of type I IFN and subsequent ISGs (104, 105) (**Figure 2**). As the implicated cGAS-STING IFN-inducing pathway also involves sensing of cytosolic DNA, mitochondrial dysfunction and release of mitochondrial DNA may be a common trigger of cell-intrinsic immune pathways downstream of inhibition of cholesterol biosynthesis or cholesterol accumulation (99, 104). Thus, innate immune pathways have evolved mechanisms to survey both accumulation of cholesterol and inhibition of cholesterol biosynthesis.

Host signaling from the plasma membrane is dependent on cholesterol content as cholesterol promotes the formation of signaling competent lipid rafts (106). Certain PRRs and cytokine receptors likely require cholesterol-dependent lipid rafts for efficient signal transduction in response to microbial infection and inflammation (106, 107). However, cholesterol presence in the plasma membrane can also be detrimental to the host response as it serves as a ligand for bacterial pore-forming toxins known as cholesterol-dependent cytolysins (CDCs) (90, 105). Moreover, some bacteria and viruses utilize cholesterol or related receptors, such as LDLR, for entry into the host (3, 59, 91). Thus, the regulation of production, import, storage, and export of cholesterol are likely under innate immune control to prevent infection (90). If pathogens or host metabolic dysfunction perturb these cholesterol control pathways beyond the Goldilocks zone of normal physiological concentrations, cell intrinsic immune responses are activated (**Figure 1**).

## PAMP and cytokine induced alteration of host metabolism for defense

Macrophages have plasticity in terms of functional polarization states with common prototypical cell states being defined as pro-inflammatory for M1-like macrophages or homeostatic and tolerogenic for M2-like macrophages (108). While this paradigm was defined with *in vitro* polarizations, tissue-resident macrophages or recruited macrophages likely adopt a wider breadth of functional states along a continuum *in vivo* (109). However, these classifications are useful for describing the intersection of PAMP- or cytokine-induced changes to host metabolism, and their potential effects on host defense or microbial replication (108).

As stated above, many host-adapted microbes have evolved mechanisms to interact with host cell lipids and sterols such as cholesterol (90, 91). For example, CDCs can intoxicate cells and damage membranes potentially leading to cell death of innate immune cells (90, 105). PRRs and cytokine receptors are thought to impact plasma membrane cholesterol content through distinct mechanisms. TLR2, TLR7, and TLR9 signaling through MyD88 in myeloid cells can increase cholesterol import and biosynthesis (105). This process of cholesterol import occurs through activation of the protein Akt and mTORC2, which can synergize with SREBP2 (105, 110). As described above, transcriptional activity of SREBP2 results in increased cholesterol uptake and biosynthesis (95, 97). PRRs that stimulate the production of type I IFNs can also modulate cholesterol localization and production (90). Type I IFN signals through IFNAR1 and IFNAR2 to recruited kinases Tyk2 and Jak1/2 to activate the transcription factors STAT1 and STAT2 (111). STAT1/2 heterodimers can downregulate genes involved in cholesterol biosynthesis and cholesterol import, while also allowing for removal of cholesterol from cellular membranes through upregulation of the enzyme cholesterol 25-hydroxylase (Ch25h) (111–113). Ch25h can produce the metabolite 25-HC from cholesterol (90). 25-HC mediates several events that result in decreased cholesterol content of cellular membranes by activating the ER resident enzymes ACAT1 and ACAT2 to esterify cholesterol for storage in lipid droplets (90, 95, 97). 25-HC also can inhibit the activity of SREBP2 thus downregulating cholesterol synthesis and uptake (90, 97). Type I and type II IFN signaling on host cells can result in cholesterol esterification and relocation from cellular membranes that is protective in the context of CDC treatment (105). Moreover, 25-HC treatment alone can result in depletion of cholesterol from host cell membranes that results in inhibition of viral entry (112, 113). While alteration of cholesterol homeostasis is a conspicuous outcome of IFN signaling, IFNs can also reprogram host cell metabolism in other pleiotropic ways including increased fatty acid oxidation, oxidative phosphorylation, and expression of ISGs that may impact cell-intrinsic immunity and pathogenesis (114, 115).

**Figure 3:**
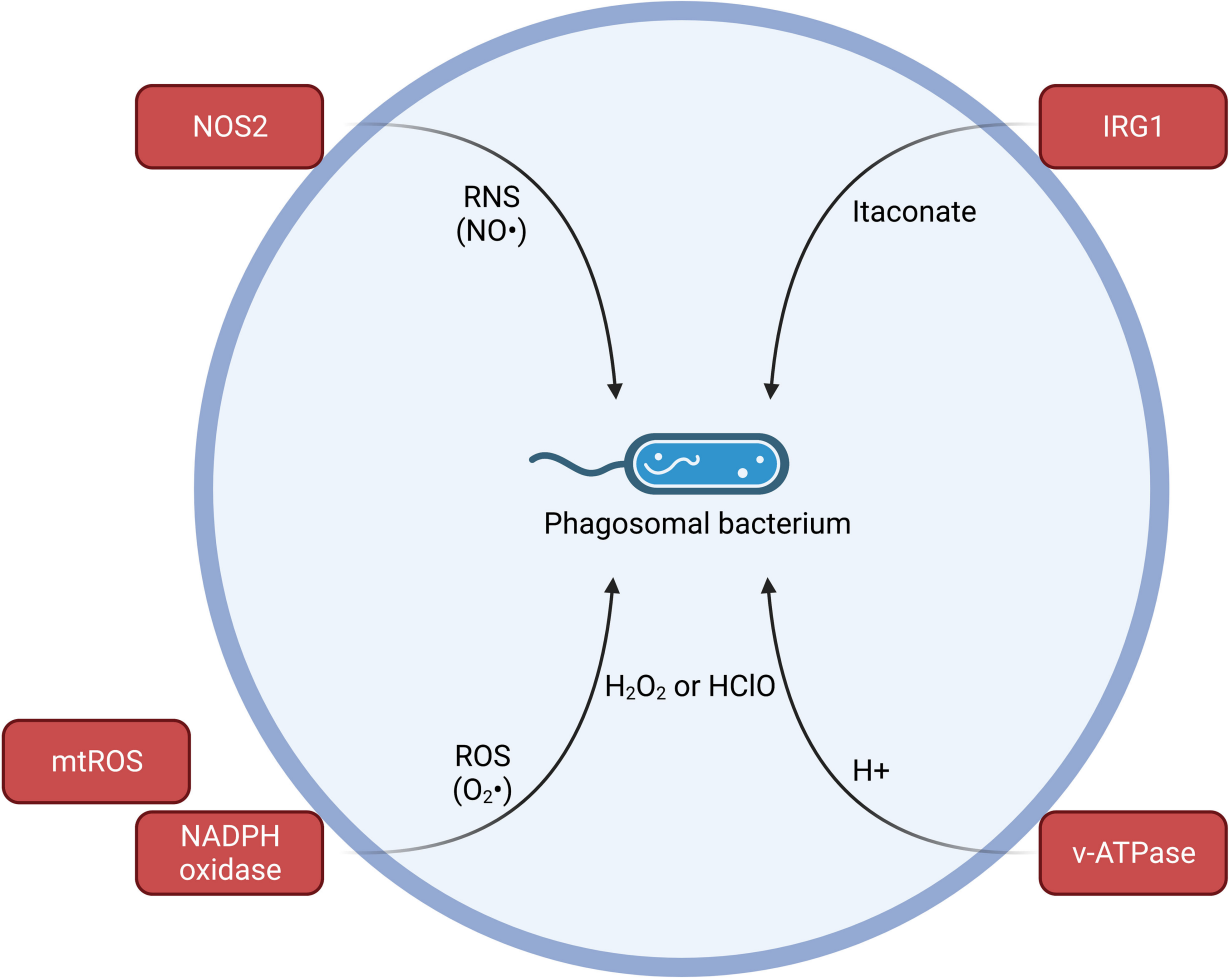
Host-adapted delivery of potentially toxic metabolites for control of intracellular pathogens. Sensation of PAMPs, cytokines, and IFNs can promote cell-intrinsic immune responses such as generation of antimicrobial metabolites. Upregulation of NOS2 can produce NO radicals from the amino acid Arginine. TLR driven mitochondrial ROS (mtROS) or phagocytosis- and BAI1-induced NADPH oxidase production of ROS. ROS can cooperate with low pH environment created by pumping H+ ions into the vesicle lumen *via* the action of v-ATPase to form hydrogen peroxide and hypochlorous acid. Accumulation of TCA intermediates based on break in the TCA cycle in activated macrophages allows for Irg1 production of anti-microbial itaconate that can be delivered to a pathogen-containing phagosome *via* RAB32.

Further considering a canonical M1-like cell state through the LPS-TLR4 axis in macrophages has uncovered metabolic shifts that can promote inflammation, restrict essential cofactors or nutrients, and deliver potentially toxic molecules to microbial replication sites. The enzyme NOS2 is regulated by the transcription factor NF-κB that is commonly activated downstream of pro-inflammatory cytokine receptors and many families of PRRs including TLRs (116). NOS2 can produce reactive nitrogen species (RNS), specifically NO radicals, from the amino acid arginine (108, 116) (**Figure 3**). Conversely, M2 polarized macrophages express arginase that may act to further limit anti-microbial NO production by limiting arginine levels (108). M2 macrophages are polarized through cytokines such as IL-4. M2 macrophages have metabolic shifts towards fatty acid oxidation and oxidative phosphorylation (108). Some pathogens, including *Salmonella*, replicate better in M2 macrophages that may depend on these metabolic shifts and glucose metabolism (117, 118). *Salmonella* appears able to induce or enrich for M2-like macrophages in part through action of the effector SteE (119–121). Sensing of microbial invasion by several mechanisms can also induce the production of ROS, such as superoxide and hydrogen peroxide. Downstream of TLR engagement, a TRAF6 dependent mechanism can activate ROS production from phagosome-proximal mitochondria (122). Moreover, NADPH oxidase, which sits within the phagosome membrane, can produce anti-microbial ROS downstream of phagocytosis or BAI1 sensing of bacterial LPS (123, 124) (**Figure 3**). After phagocytosis, the phagosome resident v-ATPase can also lower intra-luminal pH that can have direct and indirect anti-microbial activity, such as through combination with ROS to generate hypochlorous acid (125) (**Figure 3**). Finally, M1 polarized macrophages also express the metabolic and host defense protein Irg1 (126). M1 polarized macrophages have a break in the TCA cycle as described above through conversion of arginine into citrulline and NO, but also have an additional break in the TCA cycle that results from type I IFN responses and subsequent repression of IDH1 (127–130). IDH1 repression can lead to accumulation of citrate within activated macrophages (129, 130). As TCA cycle intermediates accumulate due to a defective TCA cycle, IRG1 can utilize aconitate to produce itaconate (126, 129, 130). Itaconate can further lead to accumulation of succinate as it acts as an inhibitor of SDH (131). Itaconate has been described to have direct anti-bacterial activities and was recently shown to be directly delivered to pathogen containing phagosomes by RAB32 (132) (**Figure 3**). Itaconate can also post-translationally modify inflammasome related proteins, such as NLRP3 and murine GSDMD, further highlighting that metabolites control cell-intrinsic immune responses and functional cell fate decisions through diverse mechanisms (133, 134).

## Microbe manipulation of the host

Virus and bacteria are expert metabolic engineers who aim to shift host metabolism towards favorable conditions for microbial replication (3, 59). While listing all the effectors and pathogens that can promote metabolic rewiring is beyond the conceptual aims of this section, we mention common and broad metabolic nodes that many microbes seem to prefer and have evolved mechanisms to induce within cells. Viral infections likely have different metabolic needs depending on the infective life cycle of the virus. For example, during lytic replication of certain viruses there is a need for increased supply of nucleotides, amino acids, and fatty acids for quick replication (59). Conversely, during latent viral infections there is less demand on nutrients and an emphasis on infection of naturally long-lived cells, such as neurons, or viral strategies to prolong cell viability in the case of oncogenic viruses (3, 59). Bacteria also manipulate their hosts to promote metabolism that benefits replicative needs (3, 91).

A major axis that is targeted by viruses and bacteria alike is the mTOR pathway as we have already discussed with specific inputs such as amino acid availability and control of mTORC1 activity (57, 61). Promoting the PI3K and Akt arms of mTOR activation maintains translation and cell growth while inhibiting apoptotic cell death (61). Moreover, upregulation of glucose uptake can promote mTOR activity (61). AMPK senses host energetic state by surrogate through monitoring AMP levels. AMPK negatively regulates mTORC1 in response to high AMP to ATP ratios (61). Thus, modulation of AMPK activities is also a strategy that viruses and bacteria can use to promote pro-microbe metabolic shifts (3, 59). Many pattern recognition receptor and cytokine receptor pathways can activate mTORC1 and mTORC2 which promotes production of conventionally secreted pro-inflammatory cytokines and IFNs. For example, innate immune responses to HSV-1 infection require mTORC2 and mTORC1 downstream of TLR3 signaling for optimal IFN production and viral suppression (135). As discussed above, autophagy may directly and indirectly inhibit viral and bacterial infective life cycles through capture of microbes during xenophagy (69).

As mentioned above, oxidative phosphorylation and fatty acid oxidation can naturally be promoted within IL-4 stimulated (M2) macrophages (108). Consequently, several viruses and bacterial pathogens replicate well within cells that have these metabolic parameters likely because fatty acid oxidation results in generation of acetyl-CoA (3). Acetyl-CoA, in cells with an intact TCA cycle, can generate reducing equivalents such as NADH and FADH_2_ that promote oxidative phosphorylation (108). Other specific metabolic alterations induced by microbes exist that may represent tissue-niche or pathogen-specific needs for replication or dissemination. More work is needed to delineate host metabolic manipulation by microbial effectors for the purposes of promoting cell survival during replication or promoting different forms of cell death during dissemination. As such, microbial gene regulation may change over the course of the infective life cycle in response to metabolic cues that represent tissue and cellular location or more broadly metabolic status of the host.

## Metabolite modulation of virulence programs

After detection of invading pathogens, the host can adaptively create harsh metabolic environments for microbial clearance (**Figure 3**). However, prior to infection, host-derived metabolites in the tissue microenvironment influence the ability of microbes to enact virulence programs and further nutrient acquisition (1). Here, we provide prototypical examples of how nutrient availability controls bacterial virulence programs for enteric microbes, but analogous circuits may exist in other tissues and microbes. Moreover, recent work has suggested that metabolic mutualism may be an alternative strategy to host defense by promoting tolerance and outgrowth of less virulent strains (136).

The aim of nutritional immunity is to restrict required cofactors or metabolites to limit colonization and growth of bacteria (137). Canonical nutritional immunity has largely focused on regulation of essential transition metal ions required for bacterial viability. We consider iron availability as a prototypical example of host-evolved nutritional immunity but suggest that nutritional immunity likely extends to energy sources as well such as glucose, amino acids, and lipids. Seminal studies of the iron sequestering activity of transferrin provided the first conceptual example that host-related depletion of iron could affect microbial replication or viability (138, 139). Iron metabolism is now appreciated to include other mechanisms of movement and storage that may depend on the cellular or subcellular location of microbial infection. One iron sequestering circuit involves the production of hepcidin during infection or inflammation that depends on pro-inflammatory cytokine signaling in the liver (140). Hepcidin can subsequently induce internalization of the iron transporter ferroportin (140). Lowering intracellular iron through the use of hepcidin inhibitors or genetic deficiency for homeostatic iron regulator protein (HFE) also augments intracellular growth of *Salmonella* and blunted TLR4 signal transduction in macrophages (141). In circulation, activated neutrophils can produce lactoferrin, a related iron sequestering protein to transferrin (142). On the subcellular level, macrophages can express the iron transporter NRAMP1 to restrict iron in the lumen of vesicles, such as the phagosome, to limit access to invading pathogens (143, 144). These strategies represent systemic, tissue, and subcellular mechanisms by which the host can adaptively modulate the concentration of iron that is available to microbes.

*Salmonella* can cause gastroenteritis or systemic inflammation depending on host genotype and infection site (1). As *Salmonella* often resides within the gastrointestinal tract, host nutrient availability may control virulence programs. One experimental method utilized in the laboratory to imbue *Salmonella* with an invasive phenotype is anoxic and high salt growth conditions (145, 146). Growth under these conditions increases expression of the *Salmonella* pathogenicity island 1 (SPI-1) that encodes type 3 secretion system components and corresponding injected effectors (145, 146). This circuit may instruct the bacterium to upregulate an invasion phenotype through virulence factors when environmental conditions in the gut are suitable for infection. Notably, the host has evolved strategies to sense virulence injection machinery and structurally related motility machinery through the action of the NAIP : NLRC4 inflammasome (6). Thus, host environmental and nutrient status can influence bacterial virulence programs, but the host has also evolved strategies to guard against these required tissue invasion proteins, including the type III secretion system and flagellin. Similarly, other microbes have evolved distinct nutrient cues and virulence programs related to their tissue nice and infective life cycle.

As introduced above, iron sequestration is another major host defense strategy in nutritional immunity. Provocatively, a recent report has described tolerance schemes may serve to limit *Citrobacter rodentium* virulence in the context of enteric infection in mice (136). By analyzing surviving mice after a lethal dose 50 experiment, iron metabolism was identified as a determinant of morbidity. Supplementation of iron during a normally lethal dose 100 was able to confer protection from lethality and reduce gut pathology. Specifically, iron supplementation reduced expression of virulence genes in *C. rodentium* through a microbe circuit dependent on glucose availability (136). Thus, nutrient supplementation may also be a strategy to reduce virulence of pathogenic bacteria as an alternative to sterilizing host defense. This mutual metabolic interaction and host tolerance scheme may be particularly useful under conditions where nutritional restriction leads to upregulation of virulence programs or host defense programs lead to a high degree of collateral damage as could lead to organismal demise. Selection of less virulent strains through host-derived metabolites may provide a framework for delineating commensalization of host-associated microbes.

Interspecies competition for nutrients can also cue pathogens for increased virulence (1). A pathogenic strain of *Escherichia coli*, known as Enterohemorrhagic *E. coli* (EHEC), activates expression of its enterocyte effacement pathogenicity associated island in response to nutrient signals (147–149). EHEC is primarily thought to be an extracellular pathogen that interacts with an infected host cell through adherence and modulation of nutrient trafficking and cytoskeletal elements (150). The expression of adherence proteins, type III secretion systems, and virulent effectors is upregulated under limiting glucose conditions due to host nutritional immunity or in the presence of interspecies competition for similar nutrient stores (1). Providing the potentially pathogenic bacteria *C. rodentium* with glucose led to selection of less virulent strains and increased host survival in mouse models (136). This may suggest that similar nutrient-focused therapies could be employed as therapeutics during infection with other enteric pathogens such as EHEC. Alternatively, EHEC can also upregulate this virulence program in the presence of succinate (147). Recent work now suggests that during the intracellular lifestyle of *Salmonella* in infected macrophages, accumulation of succinate can also regulate virulence programs encoded by SPI-2 (151). These studies highlight the similarity in nutrient dependency or sensitivity for expression of virulence programs in microbes. These virulence circuits may have evolved even between different tissue or cellular niche to indicate host defensive adaptations or metabolic stress.

## Future directions

In this review, we posit a Goldilocks zone model for host surveillance of metabolic shifts whereby normal physiologic shifts are tolerated to provide cells with metabolic flexibility to carry out homeostatic functions. However, guard circuits and cell-intrinsic immune programs are enacted when wide perturbations occur in either direction of this Goldilocks zone. We focused this discussion on a particular class of cell-intrinsic immune pathways, namely the inflammasomes, but suggest that other circuits exist to promote direct antimicrobial metabolite production or restriction of host metabolites, such as transition metals and cholesterol. Furthermore, different cell types may have unique guard circuits and different allowable shifts in metabolism that reflect the division of labor for that cell type and the required metabolic plasticity for cellular functions and cell states. More work is needed to discover other innate immune surveillance of Goldilocks zones for specific metabolites or metabolic pathway activities.

Consequently, host-adapted pathogens have also evolved mechanisms to promote metabolic conditions that favor their biosynthetic or dissemination needs. Creation of better cellular models and investigation of metabolic status in *ex vivo* samples or *in vivo* settings will be required to map certain metabolic perturbations to pathogen needs. Moreover, there may exist mechanisms whereby modulation of metabolic pathways or specific metabolites can change functional cell fates for therapeutic benefit. We may discover avenues for metabolic means of functional cellular reprogramming through investigation of evolved effectors from pathogens. Moreover, nutritional immunity can be expanded beyond the control of a few select energy sources or transition metals to include methodologies to change microbial virulence patterns in response to environmental conditions. An attractive approach is to model commensalization for therapeutic benefit through supply or starvation of certain metabolites that control bacterial virulence patterns. Bioinformatic analysis is beginning to map the theoretical chemistries that microbial species can accomplish. These analyses may uncover unknown dependencies on host- or other microbe-derived nutrients that can be leveraged for next-generation nutritional immune interventions.

Finally, delineation of microbe-induced metabolic shifts as being beneficial to host defense or pro-microbe require comparative analysis of model microbe sensing by the innate immune system, namely through analysis of immunometabolism downstream of purified microbial ligands. Through integration of these pioneering studies with single or combinatorial ligands and infection with live pathogenic or mutant strains of microbes, we may map contributions of metabolic shifts for host defense or inflammation compared to metabolic shifts that promote pathogenesis.

## Author contributions

CE conceptualization, literature review, first draft writing, figure design, and funding acquisition. IF conceptualization, first draft writing, revision, figure design. All authors contributed to the article and approved the submitted version

## Funding

CE is supported by a Ragon Early Independence Fellowship.

## Acknowledgments

We thank Jon Kagan and Bryan Bryson for thoughtful comments and helpful discussion on themes related to this manuscript. We apologize to members of the community whose studies were not cited herein due to space constraints. Figures were generated using Biorender.

## Conflict of interest

The reviewers MG and XZ declared a shared parent affiliation with the authors to the handling editor at the time of review.

## Publisher’s note

All claims expressed in this article are solely those of the authors and do not necessarily represent those of their affiliated organizations, or those of the publisher, the editors and the reviewers. Any product that may be evaluated in this article, or claim that may be made by its manufacturer, is not guaranteed or endorsed by the publisher.
